# Facile synthesis of 3D flower-like Pt nanostructures on polypyrrole nanowire matrix for enhanced methanol oxidation

**DOI:** 10.1039/c7ra13269g

**Published:** 2018-03-14

**Authors:** E. Mazzotta, A. Caroli, A. Pennetta, G. E. De Benedetto, E. Primiceri, A. G. Monteduro, G. Maruccio, C. Malitesta

**Affiliations:** Dipartimento di Scienze e Tecnologie Biologiche ed Ambientali, Università del Salento Via Monteroni 73100 Lecce Italy elisabetta.mazzotta@unisalento.it; Dipartimento di Beni Culturali, Università del Salento Via D. Birago, 64 73100 Lecce Italy; CNR NANOTEC – Institute of Nanotechnology c/o Campus Ecotekne Via Monteroni 73100 Lecce Italy; Dipartimento di Matematica e Fisica, Università del Salento Via per Arnesano 73100 Lecce Italy.; National Institute of Gastroenterology “S. De Bellis” Research Hospital via Turi 27, 70013 Castellana Grotte Bari Italy

## Abstract

Here we report the simple and rapid synthesis of three-dimension Pt flower-like nanostructures (PtNFs) on a polypyrrole nanowires (PPyNWs) matrix. Both PtNFs and PPyNWs are prepared by an electrochemical approach without using any seed, template or surfactant. The morphology and chemical composition of the resulting PtNF/PPyNWs hybrids are characterized by scanning electron microscopy and by X-ray photoelectron spectroscopy, respectively. Taking methanol oxidation as a model catalysis reaction, the electrocatalytic performance of the as-prepared PtNF/PPyNWs system has been evaluated by cyclic voltammetry and chronoamperometry, evidencing that these 3D materials exhibit excellent electrocatalytic activity and high level of poisoning tolerance to the carbonaceous oxidative intermediates. Such electrocatalytic performances can be ascribed to the combined effect of the flower-like structure promoting the exposure of more sites and the polymer nanowires matrix endorsing high dispersion of PtNF on a high electrochemically active surface area, besides the removal of sub-products from electrocatalytic sites.

## Abstract 2

Platinum nanoflowers on polymer nanowires exhibit catalytic properties ascribed to the effect of high surface area flower-like structures and polymer 3D structure.

## Graphical abstract

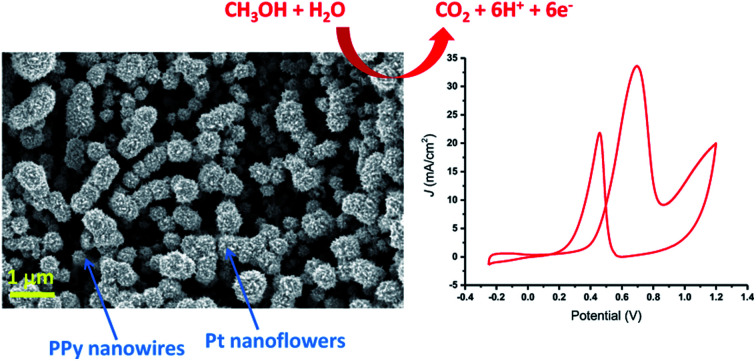

## Introduction

Platinum electrocatalytic properties are well-known since long and have stimulated extensive research leading to several applications, mainly related to its use in chemical sensors^1^ and as catalyst in the production of hydrogen from methane,^2^ in the reduction of pollutant gases emitted from automobiles^3^ and in fuel cells.^4^ It is generally accepted that both catalytic efficiency and selectivity are highly dependent on the size and shape of platinum material. In the past several years many achievements have been made in the synthesis of Pt nanocatalysts, including size-controlled^5^ and shape-controlled Pt nanostructures.^6^ Such a growing interest towards the synthesis of nanosized Pt has been encouraged also by the need of promoting a highly efficient platinum use due to its expensive nature critically limiting its technological viability.

At the present, one of the explored strategies toward this direction consists in tailoring the shape and morphology of Pt-based nanostructures, especially approaching to more catalytically active sites (corners, edges, steps, *etc.*) or more active facets.^7^ This is why, among various developed Pt nanoarchitectures, including nanoparticles,^8^ nanosheets,^9^ cauliflower-like^10^ and cubic^11^ particles, very recently nanoflowers have emerged as compelling materials due to their three-dimension structure, which provides favorable surface areas and active centers for electrocatalysis supplying enough adsorption sites for all involved molecules in a narrow space. Indeed, the synthesis of 3D Pt nanoflowers (PtNF) offers a valid chance to enhance the catalytic performance of Pt nanomaterials, as shown by the high research interest devoted to this field during the last years, leading to several successful catalytic applications of PtNF, especially in fuel cells fabrication.

Although in some cases the synthesis of PtNF has been successfully achieved directly on bare flat substrates such as glassy carbon,^10,12–16^ ITO electrodes^16–20^ and silicon substrates,^18^ an important role has been increasingly recognized to the Pt catalyst supports, used for the dispersion of catalyst particles with the aim to increase the interfacial surface area, and thus to enhance the electrocatalytic performances. Carbon based materials as carbon nanotubes (CNTs),^21^ graphene sheets,^22^ carbon paper^23^ revealed to be suitable candidate for such applications due to their unique properties as high surface area, electrical conductivity and high mechanical strength. Nonetheless, in most cases, their assembly results in carbon nano-films embedding the catalyst, having a few nanometer, not easily tunable thickness, and thus limiting the overall 3D spatial organization. In the case of CNTs, limitations in obtaining a 3D open structure are related also to their insolubility and strong tendency to aggregate in aqueous solutions.^21^ The development of novel catalyst supports for obtaining highly dispersed PtNF on a three-dimension highly open structure can thus represent a strategy for further enhancing Pt catalytic efficiency and constitute one of the major challenges in research today. Such requirement is particularly stringent in methanol oxidation based fuel cells where the removal of gaseous products could be also promoted by the use of anodes with open 3D internal structure.^5^

The assembly of nanocomposites integrating PtNF and 3D support materials has been recently proposed. Zhang *et al.*^24^ fabricated a graphene 3D foam by using a nickel foam as template and subsequently depositing Pt flower-like nanoparticles by galvanic replacement reaction between Ni and Pt. The observed enhanced catalytic behavior is induced, according to authors, by the highly dispersed Pt nanoparticles with more exposed active sites, indicating the high utilization of Pt. With the aim of improving CNT functionalization with PtNF, obtaining a controllable deposition on easily dispersible CNT in solution, Yang *et al.*^21^ adopted the polymer wrapping technique using poly(sodium 4-styrenesulfonate) (PSS) as modifying polymer. In the proposed mechanism, PSS plays a dual role: dispersing stable CNTs into solution in water, and providing functional groups that bind Pt nanoparticles. As-prepared composite systems showed a large electroactive specific surface area, and presented excellent electrocatalytic activity for oxygen reduction due to highly dispersed catalyst on the CNT support. A 3D structure integrating PtNF has been developed by Wang *et al.*^25^ who proposed the synthesis of PtNF on porous silicon by a two-step approach consisting in a physical modification of silicon surface to obtain a rough surface with high surface energy, achieved by inductively coupled plasma etching, and a chemical modification to create S–H groups acting as reducing agent, realized by HF pretreatment, involved in PtNF deposition onto porous silicon through chemical reduction. The high surface area structure thus obtained was responsible for the excellent electrocatalytic activity for methanol oxidation reaction.

In the panorama of support material for Pt catalyst, also the use of conducting polymers, especially with nanosized structure^26,27^ has been successfully proposed as an effective strategy for enhancing metal electrocatalytic activity due to the high catalyst dispersion promoted by polymer nanostructure, and to possible synergistic effect involving both catalyst and support polymer.^28^ Nonetheless, to the best of our knowledge, approaches described so far are restricted to Pt nanoparticles, with the only example on PtNF integrated with polyaniline nanofiber focused on not catalytic sensing applications.^29^

In the present work, for the first time, the synthesis of Pt flower-like nanostructures is performed on a conducting polymeric support consisting of a three-dimension polypyrrole nanowires (PPyNWs) matrix, obtaining a composite material with excellent catalytic performances, tested against methanol oxidation. The improved electrocatalytic behavior is ascribed to the combined effect of high surface area flower-like structures and polymer nanowire 3D structure possibly promoting their high dispersion while keeping small charge transfer resistance and fast reaction rate due to good PPy electron conductivity.^26^ The developed PtNF/PPyNW system is assembled by a facile electrochemical approach without using any seed, template or surfactant, allowing the simple and rapid deposition of PPy nanowires and, subsequently, PtNF directly on the electrode surface with good adherence and high homogeneity. The use of a whole electrochemical approach results to be beneficial respect to chemical synthesis routes^21,25^ requiring organic ligand stabilizers which can be difficult to separate and can have a negative impact on the performance of the catalysts, and using multi-step, time consuming and complex procedures limiting applicability and large-scale production. In addition, the use of an electrochemically synthesized nanostructured conducting polymer as support material, contrarily to other developed 3D structure integrating PtNFs,^24,25^ offers the additional benefits of properly tuning its morphology by controlling some experimental parameters,^27^ also in relation to the desired (catalytic) applications, by using a low-cost, commercially available technology.

## Experimental

All reagents were of analytical grade and were used as received from Sigma. Ultrapure water (Millipore Milli-Q, 18.2 MΩ cm^−1^) was used.

Electrochemical experiments were carried out with a CHI 660D Potentiostat (CH Instruments) controlled by computer. A one-compartment three electrodes cell was used, consisting of a glassy carbon (GC) disc (diameter 3 mm), a platinum wire and a saturated calomel electrode (SCE) as working, counter and reference electrode, respectively. All applied potential values are referred to SCE. Before use, the GC disc surface was polished successively with 1.0 μm-, 0.3 μm- and 0.05 μm alumina slurry, and then ultrasonically rinsed twice with copious amounts of water and ethanol.

PPyNWs electropolymerization was performed from a solution of pyrrole 0.1 M in K_2_HPO_4_ 0.2 M solution (pH adjusted to 6.8 by adding NaOH) containing LiClO_4_ 0.07 M by applying a constant potential of 0.85 V until a charge of 0.43 C cm^−2^ was circulated. PPyNWs were then cycled for 10 cycles from −0.25 V to 1.2 V in H_2_SO_4_ 0.5 M, scan rate 50 mV s^−1^ with the aim to obtain similarly electroactive PPyNW samples, thus reducing possible inter-electrode variability.

PtNFs deposition on PPyNWs was carried out by applying a constant potential of −0.25 V for 15 min and 60 min in H_2_SO_4_ 0.5 M containing H_2_PtCl_6_ 10 mM (samples denoted as PtNF/PPyNW). For a comparison, Pt was deposited also under the same experimental conditions by pulsed potential by applying a two-step potential program (−0.25 V for 10 s, 0.4 for 100 s) repeated 100 times (samples denoted as Ptclust/PPyNW).

Determination of Pt loading was carried out by inductively coupled plasma-mass spectrometry (ICP-MS) (X series II, Thermo Fisher Scientific) after dissolution with aqua regia (HCl 37%/HNO_3_ 70%, 3 : 1 v/v). The solution was diluted with MilliQ water and thallium (used as internal standard) was added before analysis. The isotopes monitored were ^195^Pt and ^205^Tl and external five points calibration curve with internal standard correction was used for quantification of platinum.

Methanol electrooxidation was carried out in H_2_SO_4_ 0.5 M containing CH_3_OH 1.0 M, preliminarily deaerated with N_2_ for 15 min to eliminate dissolved O_2_, by cyclic voltammetry (CV) between −0.25 V and 1.2 V at scan rate 50 mV s^−1^ and by applying a fixed potential of 0.7 V until stable current values are reached.

The morphological analysis was carried out by Scanning Electron Microscopy (SEM, Carl Zeiss Merlin). Specifically, SEM images were acquired through an in-lens detector for secondary electrons in top view configuration, with an accelerating voltage of 5 kV.

XPS measurements were performed using an Axis ULTRA DLD Spectrometer (Kratos Analytical, UK) with a monochromatic Al Kα source operating at 225 W (15 kV, 15 mA). For each sample a widescan spectrum is acquired in the binding energy range 0–1200 eV with a pass energy of 160 and 1 eV step, while high-resolution regions were acquired with a pass energy of 20 and 0.1 eV step. In both cases, the area of analysis is about 700 × 300 μm^2^. The base pressure in the instrument was 1 × 10^−9^ mbar. Data analysis was performed by CasaXPS software (version 2.3.16). Surface charging was corrected considering adventitious C 1s (BE = 285 eV).

Obtained PtNF/PPyNWs systems were characterized electrochemically by CV in H_2_SO_4_ 0.5 M between −0.25 V and 1.2 V, scan rate 50 mV s^−1^ for the evaluation and comparison of deposited platinum electroactivity. Electrochemical characterization of PtNF/PPyNWs was also performed in the same experimental conditions with different scan rates (25, 50, 100, 200, 300 mV s^−1^) and in the presence of different methanol concentrations (0.1, 0.25, 0.5, 1, 1.5, 2 M).

## Results and discussion

### Morphological characterization

**Fig. 1:**
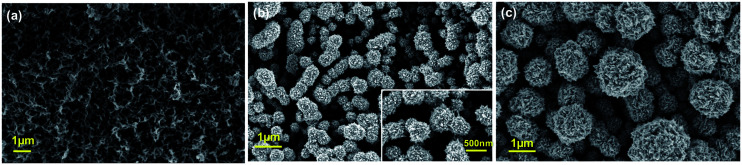
SEM top-view images of PPyNWs before PtNFs deposition (a), of PtNFs deposited on PPyNWs matrix for 900 s (b) and 3600 s (c) by applying a constant potential. Higher resolution image is provided in the inset of panel (b). See Experimental for PtNF deposition experimental conditions.

**Fig. 2:**
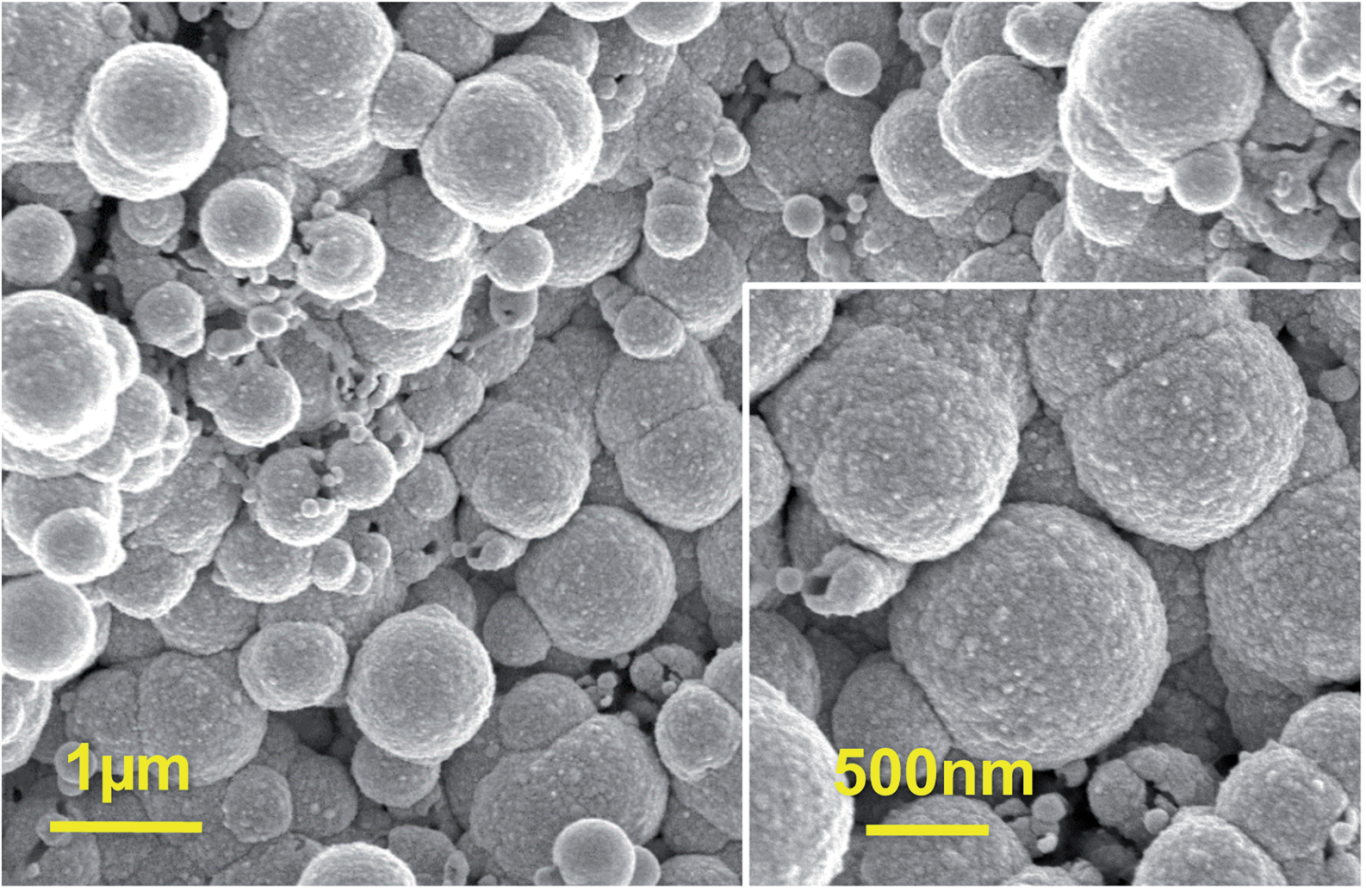
SEM top-view images of Pt clusters deposited on PPyNWs matrix for about 900 s by applying a pulsed potential. Higher resolution image is provided in the inset. See Experimental for Pt cluster deposition experimental conditions.

Fig. 1a reports SEM image of PPyNWs alone before PtNF electrochemical deposition: the high-density PPyNW network with open 3D structure can be easily observed. Fig. 1b and inset view show low and high-magnification SEM images of PtNFs electrochemically deposited for 900 s on PPyNW matrix. It can be immediately observed that Pt deposit has a flower-like morphology with a nearly uniform size (250–500 nm) containing many nanosheets as building blocks, with size of few nanometers (Fig. 2a, inset), and appears to be homogeneously distributed on PPy nanowires. In some areas of the sample, the underlying polymeric support can be still observed: the resulting morphology of the PtNF/PPyNW hybrid suggests that PtNF deposition takes place on PPy nanowire branched structure almost completely covering it. Due to its open structure (Fig. 1a), PPyNW network is highly accessible to PtNF deposition, which are thus intimately integrated with polymeric support resulting in a highly open three-dimensional structure, evidenced by the different heights at which PtNFs appears to be located. It can thus be inferred that polymer nanowires play a special role in the formation of PtNFs as, due to their open structure, solvent molecule penetrability could be promoted as well as PtCl_6_^2−^ ions diffusion.^30^ Furthermore, longer electrodeposition time such as 3600 s (Fig. 1c) produces larger size Pt structures ranging from 500 nm to about 2 μm, with flower-like morphology preserved.

With the aim to gain an insight into the formation mechanism of flower-like Pt arrays, a control experiment was performed consisting in Pt deposition on PPyNWs in the same environment but by applying a pulsed potential program with the same reduction potential (*i.e.* −0.25 V) applied for the same total interval time (*i.e.* ≈900 s) by short repeated step of 10 s, each followed by the application of 0.4 V for 100 s (Ptclust/PPyNW). Obtained SEM results are reported in Fig. 2 and inset. A completely different morphology of Pt deposits is evidently obtained consisting in almost spherical platinum cluster with diameters ranging from about 100 nm to 1 μm which partly coalesce originating higher size clusters and cover the underlying nanowire network. It can be thus drawn that the application of a constant reductive potential has a key effect in determining Pt flower-like structure. A two-staged growth model consisting in initial nucleation and subsequent anisotropic growth^31,32^ could be invoked for explaining the formation of PtNFs. A large amount of PtCl_6_^2−^ ions are quickly reduced to Pt atoms (Pt^0^) at the very early stage, leading to random formation of many tiny Pt nuclei well distributed on PPyNWs surface. Meanwhile, the newly formed Pt nuclei are preferred to grow on the firstly deposited ones instead of heterogeneous substrate. It is well known that growth rates vary in different crystallographic direction, with the fast growth plane determining the final morphology.^31^ It could be hypothesized that sulfuric acid anions HSO_4_^−^ and SO_4_^2−^ play an important role in the growth process under the adopted experimental conditions as they could be preferentially adsorbed to a certain facets of Pt crystals.^12,19,33,34^ As a consequence of the “selective” interaction between sulfuric acid ions and the facet of Pt crystals, the surface energy of the plane could be lowered and the crystal growth perpendicular to this plane could be hindered, resulting in a flower-like Pt morphology. This formation mechanism of Pt crystals could be made possible by the application of a proper constant reduction potential and could be maintained also with the increase of deposition time leading, as reported above, to the size increase of resulting Pt crystals preserving flower-like nanostructure. An evidence supporting this assumption comes from the analysis of amperometric curves relevant to PtNFs deposition (result not shown) showing a rapid current decrease at the very initial stage followed by current increase up to a final plateau current value, which is the characteristic electrodeposition behavior ascribed to initial instantaneous nucleation with subsequent progressive growth.^32^

The overall picture is completed by considering the contribution of another mechanism influencing Pt electrodeposition and possibly determining the observed differences between deposition under constant and pulsed potential. It should be taken into account that during the electrodeposition process, the mass transfer of Pt ions through the diffusive layer and the reduction reaction on the modified electrode coexist: Pt ions are consumed due to reduction process while diffusion of Pt ions from the bulk solution compensates the depletion. It is well known that when Pt ion consumption rate is higher than the diffusion rate, a Pt ion depletion layer is formed, possibly affecting the growth of Pt nuclei.^17^ It could be hypothesized that, under the adopted conditions for Pt electrodeposition (*i.e.* when the reductive potential −0.25 V is applied constantly for long interval time as 900 s), the consumed Pt ions cannot be timely compensated with the result that Pt deposition process is controlled by the diffusion process. In this case, Pt nuclei tend to grow according to the mechanism proposed above, leading to the formation of flower-like and nanosheet-like morphologies. On the other hand, in pulsed deposition, the application of anodic potential would decrease the thickness or even eliminate the Pt depletion layer, thus greatly facilitating Pt ion transport. In particular, when the duration of cathodic half-cycle (10 s) is relatively short in comparison with anodic one (100 s), the consumed Pt ions can be timely compensated during the anodic step with the result that the growth of Pt deposits is dominated by the reduction process and the formed Pt particles exhibit quasi-spherical morphology. In this case, concentration of sulfuric acid ions adsorbed to the newly formed platinum nuclei is low, determining Pt nuclei isotropic growth and leading to the formation of spherical particles without preferential growth on certain Pt crystal facets.^31^

Finally, from the above discussed results, it can be inferred that the synthesis of PtNFs is allowed by two synergic contributions from the polymeric nanowire support and from the applied potential program, both of them playing an important role in determining the achieved structure of the catalyst.

### XPS characterization

**Fig. 3:**
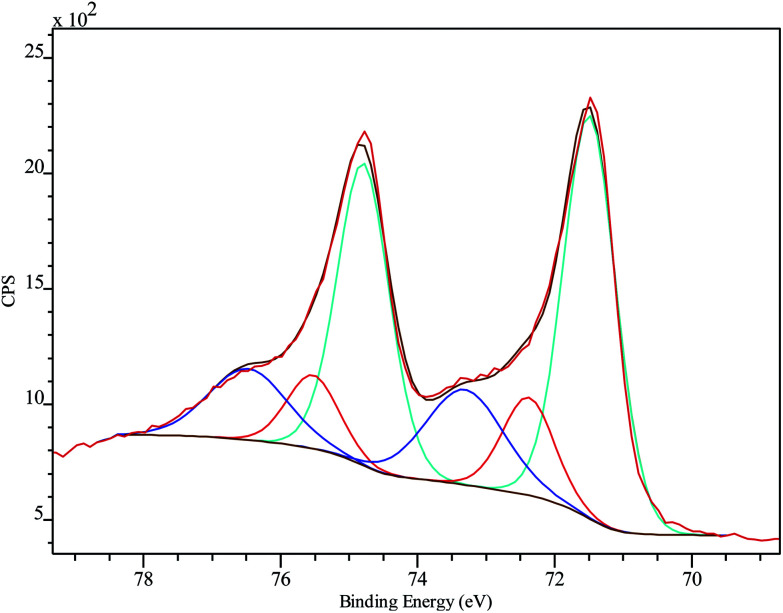
Pt 4f high-resolution XPS spectrum on PtNF/PPyNW with curve fitting results. Spectra are charging corrected.

XPS was employed to analyze the chemical composition and status of the hybrid system PtNF/PPyNW. A Pt percentage surface concentration of 0.7% was estimated. Fig. 3 presents detailed spectrum recorded in Pt 4f region and clearly shows two main peaks at 71.5 and 74.8 eV, corresponding to Pt 4f_7/2_ and 4f_5/2_, respectively. In detail, investigated system appears to be characterized by three chemically different Pt species, corresponding to metallic Pt(0) with BE of 71.5 and 74.8 eV, oxide Pt(ii) with BE of 72.4 and 75.5 eV, and oxide Pt(iv) with BE of 73.3 and 76.5 eV, respectively. The electrodeposited Pt species are predominantly in the metallic state. The presence of a small amount of oxidized Pt species (Pt(ii) and Pt(iv)) with the peak area of about 37% may be ascribed to the incomplete reduction of the Pt species.

### Electrochemical characterization. Study of the crystalline features

**Fig. 4:**
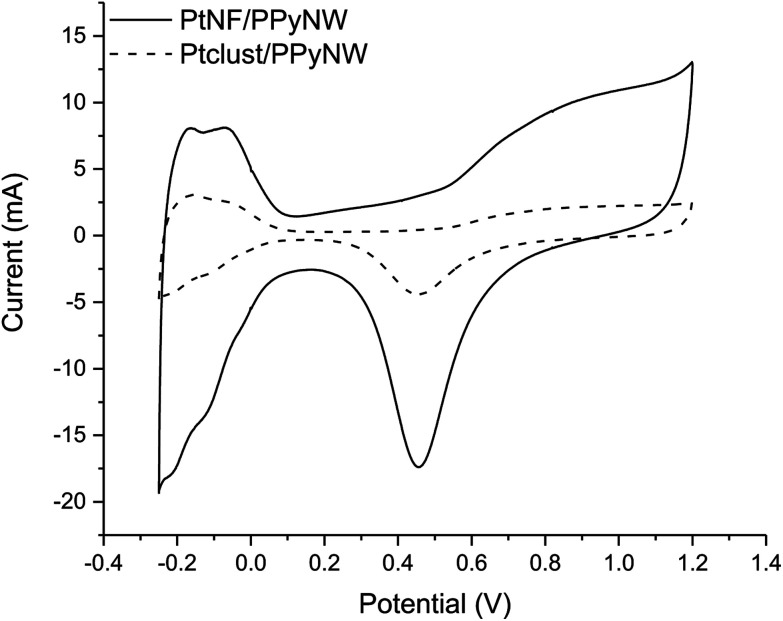
Cyclic voltammograms on PtNF/PPyNW (solid curve) and Ptclust/PPyNW (dashed curve) electrodes in 0.5 M H_2_SO_4_ solution. Scan rate: 50 mV s^−1^. Reported current values are normalized to Pt loading.

CV studies in H_2_SO_4_ solution were carried out on PtNF/PPyNW samples in order to investigate the electrochemical behavior and to understand the nature of Pt crystalline phases, by monitoring the hydrogen adsorption/desorption CV profiles.^12^ For a comparison, CV electrochemical characterization was performed also on Ptclust/PPyNWs. Fig. 4 shows CV curves recorded on both investigated systems in 0.5 M H_2_SO_4_ at a scan rate of 50 mV s^−1^. PtNF clearly shows two well-defined hydrogen adsorption peaks at about −0.16 V and −0.07 V, followed by a broad peak at higher potential value ascribed to Pt oxidation and a reduction peak at 0.45 V in the reverse scan. The observed hydrogen desorption peaks are in agreement with literature data^12,19,32^ and could be attributed to the surface adsorption characteristics of Pt (111) and Pt (110) facets, respectively, thus indicating that as-prepared PtNFs have polycrystalline nature. The comparison of CV profiles recorded on PtNF/PPyNW (solid curve) and Ptclust/PPyNW (dashed curve) suggests higher facets exposure on the surface of nanoflower-based systems. Nonetheless, significantly lower current values were recorded in all the investigated potential range on Pt clusters, with much lower hydrogen adsorption peaks between −0.25 V and 0.1 V. For a proper evaluation of electrochemical performances of two systems, the electrochemically active surface area (ECSA) was estimated by determining the area of hydrogen desorption after subtraction of double-layer region. The ECSA was evaluated according to eqn (1).1
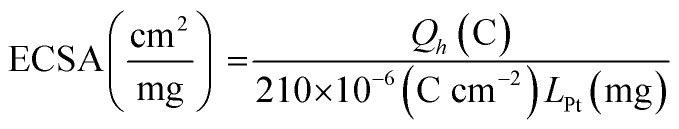
where *Q*_H_ (C) is the charge exchanged during the hydrogen desorption on the Pt surface and 210 × 10^−6^ (C cm^−2^) represents the charge required to oxidize a monolayer of hydrogen on a smooth polycrystalline Pt surface. *L*_Pt_ represents the Pt loading as determined by ICP-MS measurements. Obtained *L*_Pt_ and ECSA values for the two investigated systems are listed in Table 1. It can be noted that on Ptclust/PPyNW, a much higher amount of platinum is deposited, probably due to the regeneration of a high Pt gradient concentration at each switching potential step, promoting Pt deposition from bulk solution to electrode-solution interface. The higher ECSA value determined for PtNF/PPyNW can be ascribed to flower-like morphology which determines a significant increase of surface area providing a higher number of active sites for (catalytic) reaction. Electrochemical data are in agreement with SEM results as they confirm that, under the application of a constant reductive potential, although a minor amount of Pt is deposited, it results to be highly dispersed determining – together with the resulting flower-like morphology – an enhanced electrochemical activity, contrarily to pulsed deposition which leads to the loading of higher amount of platinum, evidently characterized by high irreproducibility probably related to agglomeration effect, extremely detrimental for electroactivity.

**Table 1:** Electrochemical parameters for methanol oxidation on PtNF/PPyNW catalysts. Reported average values with standard deviation are referred to three replicates on three different samples

Catalyst	Pt loading (mg)	ECSA (cm^2^ mg_Pt_^−1^)	Mass activity (mA mg_Pt_^−1^)
PtNF/PPyNW	0.016 ± 0.000	6.10 ± 0.79	177.27 ± 11.76
Ptclust/PPyNW	0.141 ± 0.085	4.18 ± 1.25	79.03 ± 24.60

### Methanol oxidation

**Fig. 5:**
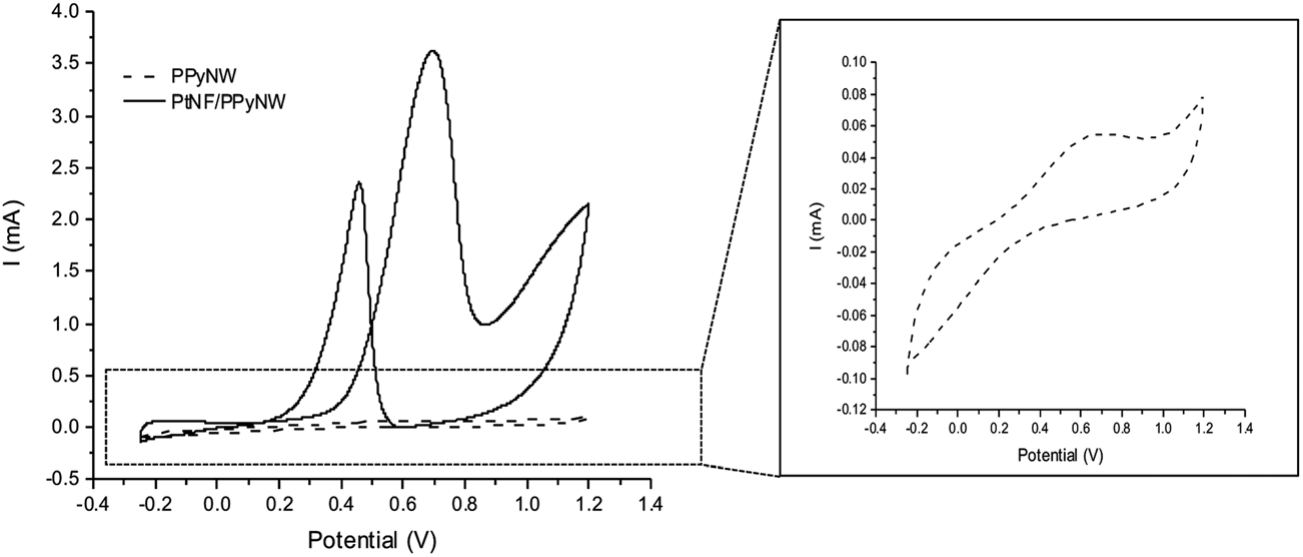
Cyclic voltammograms on PtNF/PPyNW (solid curve) and PPyNWs alone (dashed curve) in 1 M methanol solution in 0.5 M H_2_SO_4_. A magnification of CV curve on PPyNWs is reported in the inset.

The developed PtNF/PPyNW systems exhibit catalytic activity, evaluated against methanol electrochemical oxidation. Obtained CV results are illustrated in Fig. 5 showing also CV curves for methanol oxidation on PPyNWs alone for comparison. It is evident that, as expected, much lower currents are recorded on PPyNWs with no oxidation peak ascribable to methanol oxidation in forward and reverse scan, confirming that polymeric support is not involved in methanol oxidation process, which is instead completely due to PtNF catalytic effect.

**Fig. 6:**
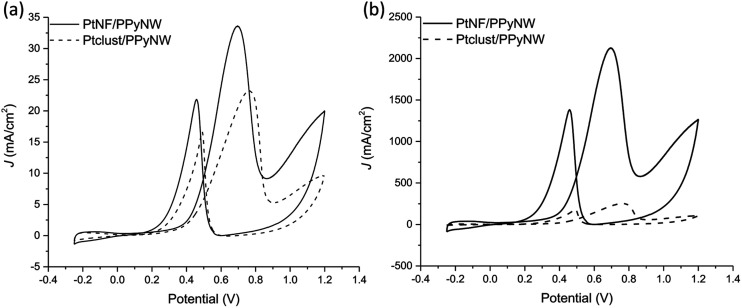
(a)–(b) Cyclic voltammograms on PtNF/PPyNW (solid curve) and Ptclust/PPyNW (dashed curve) electrodes in 0.5 M H_2_SO_4_. In particular, in panel (b) currents normalized per electrochemically active surface area (ECSA) are reported.

Fig. 6 reports the comparison between CV curves on PtNF/PPyNW (solid curve) and Ptclust/PPyNW (dashed curve). An oxidation peak is clearly observed in CV on PtNFs (solid curve) in the forward sweep at 0.69 V due to methanol oxidation, followed by another oxidation peak at 0.46 V in the backward scan, which is assigned to the secondary oxidation of carbonaceous intermediates (adsorbed CO or CO-like species generated by the incomplete oxidation of methanol) into CO_2_.^31^ It is evident that PtNFs exhibit significantly enhanced current densities compared to that of Ptclust, along with an appreciable negative shift of both forward and reverse peak potential, which on Ptclust are centered at 0.76 V and 0.49, respectively. These results clearly indicate the higher electrocatalytic activity of PtNF/PPyNW system suggesting that the flower-like morphology of Pt catalysts not only provides higher surface area but also an improved exposition of active planes/sites thereby determining a significantly enhanced electron transfer rate.^24^ The superior catalytic performances of PtNF in comparison with Ptclust are further evidenced from the comparison between ECSA–normalized current values (Fig. 6b) due to the lower amount of deposited Pt in the case of nanoflower-like structures thus indicating a more efficient use of catalyst. The ratio between the forward and backward anodic peak current density (*j*_f_/*j*_b_) is commonly used to characterize the tolerance to carbonaceous oxidative intermediates accumulation on the electrocatalyst surface. A high *j*_f_/*j*_b_ ratio implies effective methanol oxidation to the end products during the anodic scan with subsequent low accumulation and/or effective removal of the poisoning species from the electrocatalyst surface.^12^ A higher *j*_f_/*j*_b_ value is estimated for PtNF (1.46) in comparison with Ptclust (1.28), confirming better catalytic performances, also in terms of less accumulation of carbonaceous intermediates and higher tolerance towards CO poisoning.^32^ Furthermore, a role of PPyNW matrix on inhibition of the poisoning effect on Pt catalyst could be invoked^35^ due to the presence of adsorbed water or hydroxide on polymer surface, which could facilitate the oxidation of CO^12^ thus hindering the formation of strongly adsorbed poisons. In the case of Ptclust, such an effect could be minimized by the presence of highly agglomerated Pt clusters almost completely covering underlying polymeric structure, contrarily to PtNF/PPyNW system, where the open 3D structure of polymeric nanowire matrix results highly exposed to adsorption of water and hydroxide. Mass-normalized activity, which is another important parameter influencing electrocatalytic efficiency and Pt utilization,^31^ was also evaluated (Table 1), obtaining for PtNF a significantly higher value in comparison with Ptclust system, characterized by higher reproducibility (RSD 6.6% and 31.1% (*n* = 3) for PtNF and Ptclust, respectively). This results further confirms that the proposed system not only exhibits higher catalytic performances but it allows also a highly efficient platinum use, thus satisfying the need of reducing platinum consumption, which is a critical issue to be considered in the design of such catalytic systems.

**Fig. 7:**
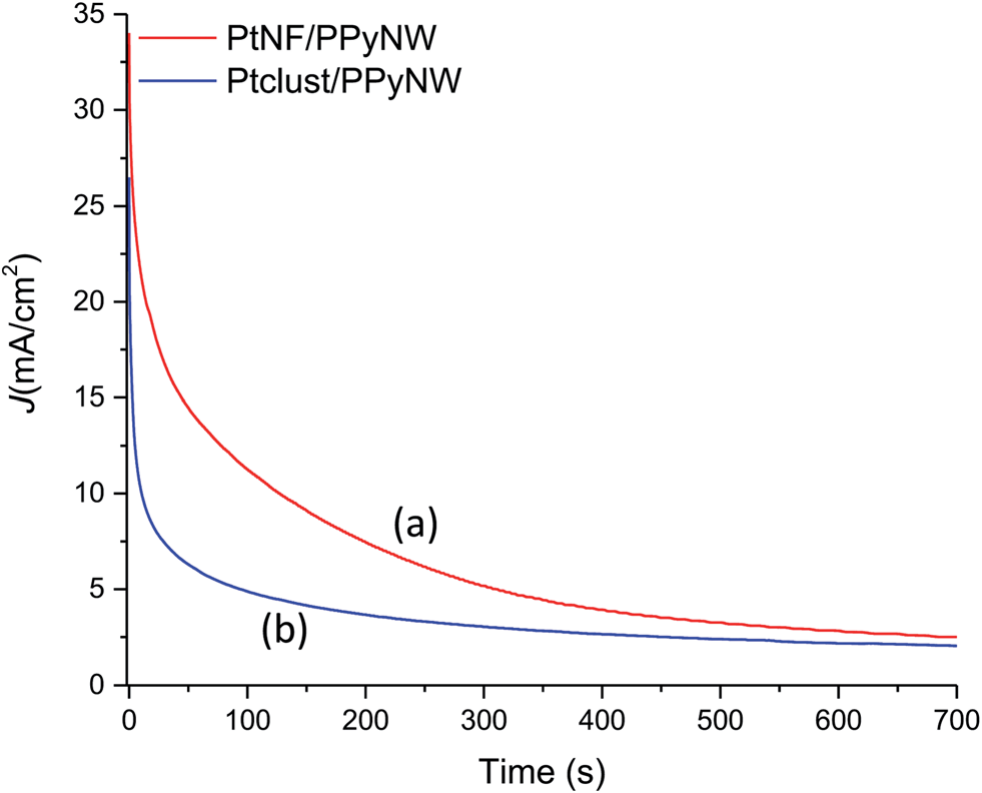
Chronoamperometric curves of PtNF/PPyNW (curve a) and Ptclust/PPyNW (curve b) for methanol oxidation at 0.7 V in 1 M methanol solution in 0.5 M H_2_SO_4_.

To further assess catalytic activity of PtNF/PPyNW in methanol oxidation, chronoamperometry tests are carried out at 0.7 V. As given in Fig. 7, the currents decay over time in a parabolic style, with a sharper decrease during the earlier minutes followed by a much slower current attenuation, reaching of a quasi-stationary within 700 s. The current decay can be ascribed to the accumulation of poisoning species, mainly CO,^30^ which could adsorb on the Pt surface during long-term experiments, thereby determining the loss of activity. It is evident that PtNF/PPyNW (curve a) exhibits lower rate of current decay in comparison with Ptclust/PPyNW (curve b), with higher quasi-stationary current value (2.52 mA cm^−2^ and 2.04 mA cm^−2^ on PtNF and on Ptclust, respectively), indicating a higher tolerance to the carbonaceous species generated during methanol oxidation, in agreement with CV results. Moreover, also better electrocatalytic activity towards the electroxidation of methanol is confirmed by the higher current density on PtNF during the whole testing time.

The above reported results confirm that the electrocatalytic properties of the PtNF/PPyNW system tested against methanol oxidation are due to the combined effect of Pt flower-like morphology and 3D open structure of PPy polymeric matrix providing, in comparison with Ptclust/PPyNW system, larger catalytic active surface area and faster electron transfer, inhibiting the undesired agglomeration of active sites and minimizing poisoning effect by intermediated species of methanol oxidation process. Moreover, the developed system exhibits enhanced or similar electrocatalytic performances in comparison with most of Pt nanoflower-based catalysts applied in methanol electroxidation, in terms of oxidation potential, *j*_f_/*j*_b_ values^10,12,14,22,24,25,36^ and mass activity.^12,31,36^

### Effect of potential scan rate and methanol concentration. Cycling stability

**Fig. 8:**
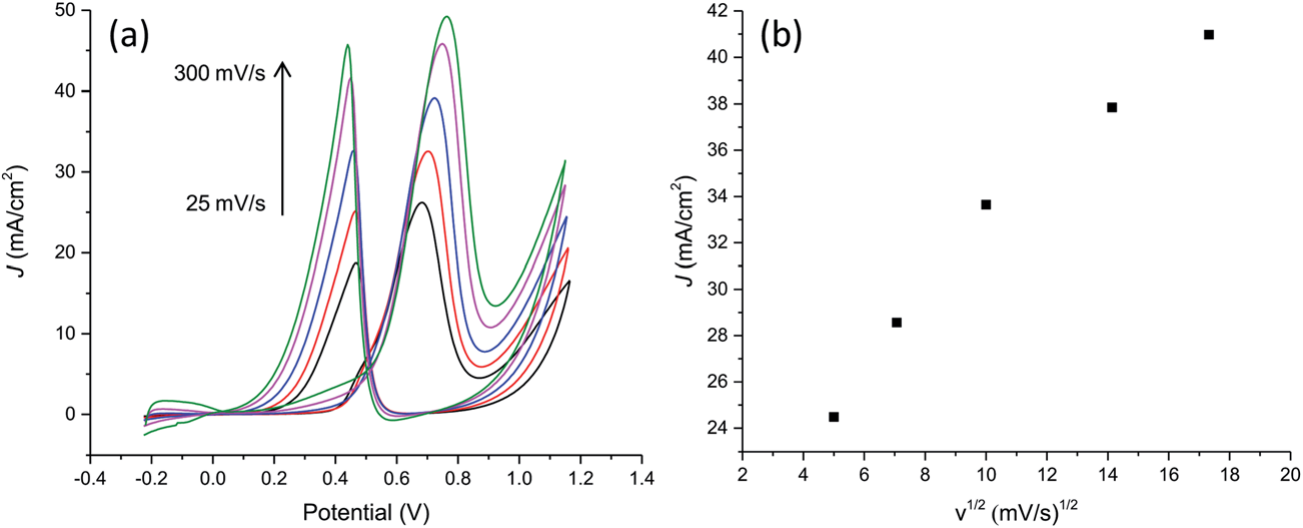
(a) Cyclic voltammograms on PtNF/PPyNW for the electroxidation of methanol at different scan rates (25, 50, 100, 200, 300 mV s^−1^) in 1 M methanol solution in 0.5 M H_2_SO_4_. (b) Plot of forward anodic peak current density *vs.* square root of scan rate.

With the aim to further characterize the electrocatalytic activity of the developed PtNF/PPyNW system, methanol transport characteristic and electrocatalytic oxidation of different methanol concentrations have been evaluated. CVs recorded in 0.5 M H_2_SO_4_ containing 1 M CH_3_OH at different scan rates are reported in Fig. 8a evidencing that the oxidation current increases when the scan rate increases. The relationship between the peak current in forward CV scans and scan rate (Fig. 8b) indicates that peak currents for methanol oxidation increase linearly with the square root of the scan rate from 25 to 300 mV s^−1^ evidencing that the electrocatalytic oxidation of methanol on PtNF/PPyNW modified electrodes is a diffusion-controlled process.^30^

**Fig. 9:**
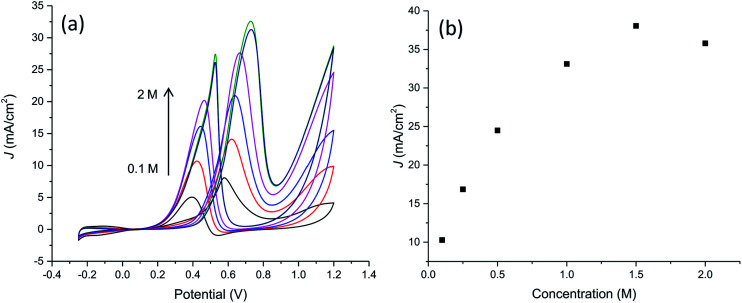
(a) Cyclic voltammograms on PtNF/PPyNW for the electroxidation of different methanol concentrations at scan rate 50 mV s^−1^ in 0.5 M H_2_SO_4_ solution. (b) Plot of forward anodic peak current density *vs.* methanol concentration.

Fig. 9a and b collects results relevant to electrocatalytic tests in the presence of different methanol concentration from 0.1 M to 2 M. It can be seen that the oxidation peak current density increases as the methanol concentration increases up to 1.5 M then exhibiting a slight drop with further increasing methanol concentration probably due to the saturation of active sites available for methanol adsorption and/or poisoning effect due to adsorbed carbonaceous intermediates. This is evidenced also by the shift of peak potential in the forward scan towards more anodic values with the increase of methanol concentration from about 0.58 V to 0.73 V in the explored concentration range, suggesting a gradually increasing inhibition of electron transfer process.

Finally, to test the long-term cycling stability of PtNF/PPyNW, the system has been continuously swept between −0.25 and 1.2 V for 300 cycles in a solution of 1.0 M methanol in 0.5 M H_2_SO_4_ recording a gradual decrease of anodic peak current density with potential cycling due to poisoning of the electrode surface, in agreement with amperometric data, with a survival of 25% compared to the first cycle after 300 scans.

## Conclusions

In the present work, the synthesis of three-dimension Pt flower-like nanostructures (PtNF) is performed on a conducting polymeric support with a three-dimension structure, consisting of polypyrrole nanowires (PPyNWs) matrix, obtaining a composite material with excellent catalytic performances in methanol electroxidation. The developed PtNF/PPyNW system is assembled by a facile and rapid whole electrochemical approach, based on the use of an electrochemically synthesized nanostructured conducting polymer as support material, offering the additional benefits of possibly tuning its morphology by controlling some experimental parameters, using a low-cost and simple technology. The obtained PtNF/PPyNW system exhibited significantly enhanced ECSA and mass activity in methanol oxidation in comparison with an analogue composite system integrating Pt clusters and PPyNWs. The observed electrocatalytic behavior is ascribed to the combined effect of high surface area flower-like structures and polymer nanowire 3D structure promoting their high dispersion while keeping small charge transfer resistance and fast reaction rate due to good PPy electron conductivity. The proposed approach thus represents a novel strategy to design catalyst and supporting material for catalysts, which further opened up promising avenues for catalytic applications as fuel cells.

## Conflicts of interest

There are no conflicts to declare.

## Supplementary Material
